# Heterogeneous nuclear ribonucleoprotein A2/B1 as a novel biomarker in elderly patients for the prediction of postoperative neurocognitive dysfunction: A prospective nested case-control study

**DOI:** 10.3389/fnagi.2022.1034041

**Published:** 2022-10-21

**Authors:** Tong Xia, Chenyi Yang, Xinyi Wang, Lili Bai, Ji Ma, Mingshu Zhao, Wei Hua, Haiyun Wang

**Affiliations:** ^1^Department of Anesthesiology, The Third Central Clinical College of Tianjin Medical University, Tianjin, China; ^2^Department of Anesthesiology, Nankai University Affinity the Third Central Hospital, Tianjin, China; ^3^Tianjin Key Laboratory of Extracorporeal Life Support for Critical Diseases, Tianjin, China; ^4^Artificial Cell Engineering Technology Research Center, Tianjin, China; ^5^Tianjin Institute of Hepatobiliary Disease, Tianjin, China

**Keywords:** postoperative neurocognitive dysfunction, biomarker, lumbar decompression and fusion, HnRNPA2/B1, Aβ42

## Abstract

**Background and objective:**

Postoperative neurocognitive dysfunction (PND) occurs in up to 54% of older patients, giving rise to the heavy psychological and economic burdens to patients and society. To date, the development of PND biomarkers remains a challenge. Heterogeneous nuclear ribonucleoprotein A2/B1 (hnRNPA2/B1) is an RNA-binding protein whose prion-like structure is prone to mutation and hence leads to neurodegenerative diseases, but its expression changes in PND remains unclear. Here, we detect the preoperative hnRNPA2/B1 level in patients with PND, and to explore its value in the prediction and diagnosis of PND.

**Methods:**

The study included 161 elderly patients undergoing lumbar decompression and fusion in Nankai University Affinity the Third Central Hospital from September 2021 to July 2022. Neuropsychological and psychometric evaluations were performed before surgery, 1 week and 3 months after surgery to diagnose the occurrence of PND, then the peripheral blood was collected from patients before induction of anesthesia. The concentration in plasma of hnRNPA2/B1 and amyloid-β 42 were determined by enzyme-linked immunosorbent assay. The median fluorescence intensity and mRNA levels of hnRNPA2/B1 in peripheral blood mononuclear cells was detected by indirect intracellular staining flow cytometry and quantitative real-time PCR, respectively.

**Results:**

The preoperative hnRNPA2/B1 level in patients with PND was higher both in short-time and long-time follow-up. We found significantly higher concentrations of hnRNPA2/B1 in PND at 7 days after surgery (median, 72.26 pg/mL vs. 54.95 pg/mL, *p* = 0.022) compared with patients without PND, and so as 3 months after surgery (median, 102.93 pg/mL vs. 56.38 pg/mL, *p* = 0.012). The area under the curve (AUC) was predicted to be 0.686 at 7 days after surgery and 0.735 at 3 months. In addition, when combining several clinical information, the diagnostic efficiency of hnRNPA2/B1 for PND could further increase (AUC, 0.707 at 7 days, 0.808 at 3 months).

**Conclusion:**

Based on the findings reported here, hnRNPA2/B1 may serve as a new and powerful predictive biomarker to identify elderly patients with PND.

## Introduction

Postoperative neurocognitive dysfunction (PND) is a cognition-related complication, whose incidence fluctuates from 20 to 54% and increases with age (Needham et al., 2017; Carr et al., 2018; Evered and Silbert, 2018). This postoperative outcome is also independently associated with some adverse effects, including increased surgical morbidity and mortality, length of hospital stay, hospitalization and out-of-hospital care costs and functional disability (Dijkstra et al., 1999; Steinmetz et al., 2009; O’Gara et al., 2020). Considering these outcomes caused by PND, it is, therefore, vital to recognize individuals prone to PND as early as possible.

Exploring PND biomarkers would help clinicians stratify patients by risk and conduct individualized management, and such biomarkers could also provide clues to help elucidate the mechanisms behind PND. Currently, the majority of the detected PND biomarkers are related to nerve damage, neurotoxicity, astrocyte injury or neurotrophic effect. One potential PND marker is neuron-specific enolase (NSE), though considered as a reliable indicator of neuronal and brain damage, but it works not very well in the early stage of brain injury (Ramlawi et al., 2006). Its diagnostic sensitivity could be reduced for its non-specificity to brain tissue, then causing a high prevalence of false-positive results and even a reverse gradient change (Rappold et al., 2016; Danielson et al., 2018). Glial fibrillary acidic protein (GFAP), a cytoskeletal protein in astrocytes and highly specific to brain, is associated with the incidence and severity of PND (Rappold et al., 2016), but Ballweg et al. (2021) found that the receiver operating characteristic (ROC) curve of GFAP to diagnose PND was only 0.49. Therefore, there remains no standard biomarkers for diagnosis and prognosis of PND.

Considering the accelerated aging of the population, incremental number of operations and consequent increasing incidence of PND, we urgently need accurate biomarkers to identify high-risk PND populations. Heterogeneous nuclear ribonucleoprotein (hnRNP) A2/B1 is an important RNA-binding protein that participate in various processes of nucleic acid metabolism, and its expression changes and mutations are closely related to neurodegenerative diseases (Zhang et al., 2021). Our previous studies have found the up-regulation of hnRNPA2/B1 in hippocampal neurons would induce the cognitive impairment of rats (Wang et al., 2020). Despite hnRNPA2/B1 has been confirmed as a significant role in the development of neurodegenerative disorders, especially amyotrophic lateral sclerosis (ALS) and Alzheimer’s disease (AD), the relationship between hnRNPA2/B1 and PND remains unclear.

It’s reported that PND shares several common molecular pathways with dementia, including neuroinflammation (Le et al., 2014; Luo et al., 2019), oxidative stress (Netto et al., 2018), impaired synaptic function (Xiao et al., 2018; Gao et al., 2021), and the microbiota–gut-brain axis (Yang et al., 2018; Jiang et al., 2019). These pathologic changes may provide physiological basis to the conversion from PND to AD (Steinmetz et al., 2009; Evered et al., 2016), the latter resulting much heavier economic burden. Therefore, we explored the hnRNPA2/B1 level in elderly patients and confirmed the potential predictive value of it to PND so that more high-risk population of PND could be identified.

## Materials and methods

### Sample size

Based on the results of hnRNPA2/B1 predictive to ALS and the reported incidence of PND, the ratio between the PND and non-PND arms was predefined in the range of 1:3 to make age and gender reveal no differences between groups, and 140 patients were presumably required for the study (α = 0.05; 1−β = 0.9). Allowing an attrition rate of 10–20%, we would recruit 161 participants finally.

### Participants

After obtaining the approval from the Ethics Committee of Nankai University Affinity the Third Central Hospital and the written informed consents from all patients, 161 consecutively admitted patients, with the age of more than 65 years, undergoing lumbar decompression and fusion within 4 h from September 2021 to July 2022 were enrolled in the study [SZX-IRB-SOP-016(F)-002-02]. The exclusion criteria were as follows: critical illness (preoperative ASA ≥ IV); history of mental illness or neurological disorders; intake of tranquilizers or antidepressants; suspected dementia or memory impairment with a score on the mini-mental state examination (MMSE) < 17 or Montreal Cognitive Assessment (MoCA) < 15; cancer; unwilling to comply with the procedures; or hearing loss.

Regarding the intraoperative anesthetic management, routine electrocardiogram, pulse oximetry, invasive blood pressure and bispectral index (BIS) were continuously monitored after the patients admitted to the operating room. All patients undergoing lumbar decompression and fusion surgery received general anesthesia with midazolam (0.05 mg/kg), sufentanil (0.3 μg/kg), etomidate (0.3 mg/kg), Cisatracurium (0.2 mg/kg), and was maintained with continuous infusion of propofol (1.2 μg/ml) and remifentanil (0.1–0.4 μg/kg/min) + 0.7 MAC sevoflurane during surgery to maintain the BIS value within 40–60. Patients under poor hemodynamic conditions should be treated timely. Additionally, all patients received postoperative analgesia *via* a patient-controlled analgesia device for the initial 48 h after surgery.

### Cognition assessment

Neuropsychological tests were assessed pre-operatively (the day before the operation), 7 days and 3 months after surgery. The battery primarily focused on memory, learning, attention, executive functions, and cognitive flexibility, and included the following tests: MMSE, MoCA, Instrumental Activities of Daily Living (IADL) as well as Clinical Dementia Rating (CDR). Mild cognitive impairment (MCI) was identified in terms of education-specific cutoff points of total scores of MMSE and MoCA, respectively. According to MMSE norms, 17∼27 for illiterate individuals, 20∼27 for participants with elementary school education, and 24∼27 for those with middle school education and above (Mitchell, 2009). MoCA score in the range of 15∼24 indicates no dementia and below the threshold for diagnosis of AD (Memoria et al., 2013) and CDR is equal to 0.5. Patients with MCI would be enrolled in the study and then followed for 3 months.

According to the definition of PND that the neurocognitive results drop over one SD from baseline when following up (Newman et al., 2001), participants were evaluated whether have PND or not and then divided into PND_1_ or non-PND_1_ according to the follow-up results of 1 week after surgery, and PND_2_ or non-PND_2_ at 3 months after surgery.

### Sample preparation

The blood samples were collected in EDTA-K2 anti-coagulation vacuum tubes before the induction of anesthesia and divided into three parts, applied to FCM, ELISA, and qRT-PCR. After centrifuging at 3,000 × g for 10 min, the plasma samples were stored at −80°C until analysis. Furthermore, the PBMCs were extracted using Lymphocyte Separation Medium (Human) (P8610, Solarbio, Beijing, China), washed with 1 × PBS (FZ1258, Solarbio, Beijing, China) 3 times totally and then flow cytometry and qRT-PCR are conducted.

### Flow cytometry

After harvesting and suspending the PBMCs, add cell fixation and permeabilization (00-5523-00, Thermo Fisher Scientific Inc., Waltham, MA, USA), anti-hnRNPA2/B1 primary antibody (ab259894, Abcam, Toronto, ON, Canada) or Rabbit Monoclonal IgG (ab172730, Abcam, Toronto, ON, Canada) as isotype control and Goat F(ab’)2 Anti-Rabbit IgG (DyLight^®^ 488, ab98507, Abcam, Toronto, ON, Canada) as the secondary antibody and incubate, respectively, at room temperature in the dark for about 30–60 min. Wash the cells 3 times by centrifugation at 400 × g for 5 min and resuspend after each incubation. Resuspend cells in 1% paraformaldehyde to prevent deterioration at last for extended storage as well as greater flexibility in planning time on the cytometer. When gating on cell populations by Flowjo data analysis software (TreeStar, USA), we would obtain the median fluorescence intensity of lymphocytes and monocytes to analyze the difference between groups.

### Enzyme linked immunosorbent assay

Concentrations of hnRNPA2/B1 (SBJ-H2321, SenBeiJia, Jiangsu, China) and Aβ_42_ (E-EL-H543c, Elabscience, Houston, USA) were examined by ELISA kit. The kit and plasma samples were balanced to room temperature half an hour before use, and the operation is fully carried out following the manufacturer’s instructions. Notably, it should be detected within 15 min after adding the stop solution. The absorbance was read on a spectrophotometer (PerklnElmer, Waltham, MA, USA) at a wavelength of 450 nm. The concentrations of hnRNPA2/B1 and Aβ_42_ were calculated according to the standard curve and presented as pg/mg protein.

### Quantitative real-time polymerase chain reaction

Total RNA was extracted from PBMC using the TRIzol reagent (15596026, Thermo Fisher Scientific Inc., Waltham, MA, USA) and its concentration was measured by spectrophotometer (Thermo Fisher Scientific, Waltham, MA, USA). The complementary DNA (cDNA) was obtained by the reverse transcription kit (RR036A, Takara, Japan) and then transcribed to mRNA using the SYBR Premix Ex Taq II (RR820A, Takara, Japan) on an ABI 7,500 instrument (Applied Biosystems, Foster City, CA, USA), with 3 duplicates set in each well. The mRNA levels were normalized to β-actin and the fold changes were calculated using the method of 2^–ΔΔCt^. The nucleotide sequences of the PCR primers (Sangon Biotech, Shanghai, China) are as follows:

β-actin mRNA: (forward 5′-CAC CAT TGG CAA TGA GCG GTT C -3′
reverse 5′-AGG TCT TTG CGG ATG TCC ACG T-3′)
hnRNPA2/B1 mRNA: (forward 5′- GCT TAA GCT TTG AAA CCA CAG A -3′
reverse 5′-CTT GAT CTT TTG CTT GCA GGA T-3′).

### Statistical analysis

Descriptive results of continuous variables were presented as the means ± standard deviations (SD) if data follow a normal distribution and homogeneity of variance, otherwise median with interquartile range (IQR). Categorical data were expressed as a percentage or counts. Intergroup comparisons were compared by the independent sample *t*-test or Kruskal-Wallis H test. For comparisons of qualitative parameters, Chi-square or Fisher exact test was applied. Multivariate logistic regression analysis was used to determine the independent risk factors of PND based on the results of previous univariate analysis. Results were shown as odds ratio (OR) and 95% CI. Additionally, we built prediction models for the occurrence of PND and then evaluated its prediction effect by the ROC curve on the basis of the results of ELISA. All statistical analysis were performed using the SPSS 25.0 software (IBM Corp. Armonk, NY, USA), and *P* < 0.05 was considered to be statistically significant.

## Results

### Patients

**FIGURE 1:**
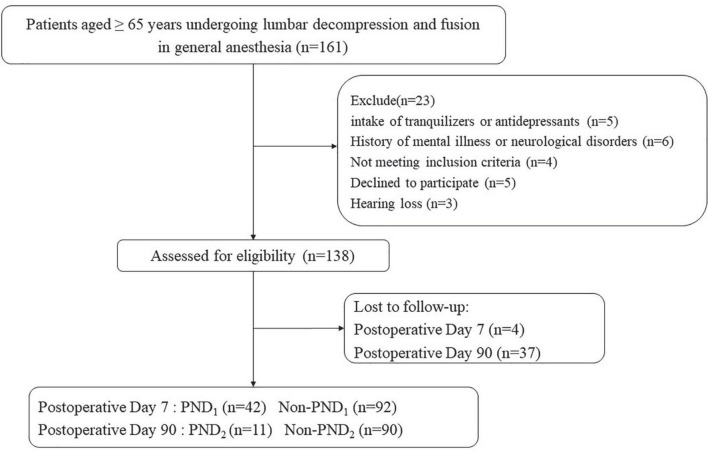
The CONSORT diagram showing patients’ flow through the study.

Among the eligible 161 patients, 138 were included in the final data analyses (Figure 1). Postoperative short-term follow-up results showed that, no clinically statistical differences were observed with regard to the gender, BMI, ASA classification, hypertension, coronary artery heart disease, and duration of anesthesia, while patients with PND presented lower education level, longer duration of surgery and were subject to receive blood transfusion during surgery compared to those without PND. Postoperative results of long-term follow-up showed no difference in baseline information between two groups (Table 1, *P* < 0.05). Moreover, pre-/post-operative scores of neuropsychological tests also displayed difference between groups (Table 2, *P* < 0.05).

**TABLE 1:** Demographic data at baseline for patients with/without PND.

	Postoperative day 7 (*n* = 134)			Postoperative day 90 (*n* = 101)		
	Non-PND_1_ (*n* = 92)	PND_1_ (*n* = 42)	*P*-value	Non-PND_2_ (*n* = 90)	PND_2_ (*n* = 11)	*P*-value
**Preoperative data**						
Male sex, *n* (%)	48.9% (*n* = 45)	40.5% (*n* = 17)	0.264	47.8% (*n* = 43)	18.2% (*n* = 2)	0.062
Age (year)	68.24 ± 4.43	68.50 ± 4.17	0.748	68.16 ± 4.36	68.27 ± 3.98	0.933
BMI (kg/m^2^)	25.61 ± 3.12	24.96 ± 3.25	0.273	25.38 ± 2.87	24.24 ± 4.32	0.246
ASA (II/III), *n*	50/42	26/16	0.413	50/40	8/3	0.277
Education level (year)	9.01 ± 4.80	7.26 ± 4.58	0.049*	8.24 ± 4.90	6.27 ± 4.73	0.210
**Comorbidity, before operation, *n* (%)**						
Hypertension	53.3% (*n* = 49)	50.0% (*n* = 21)	0.726	52.2% (*n* = 47)	72.7% (*n* = 8)	0.197
Diabetes	20.7% (*n* = 19)	38.1% (*n* = 16)	0.033*	22.2% (*n* = 20)	36.4% (*n* = 4)	0.298
Coronary Artery Heart Disease	20.7% (*n* = 19)	9.5% (*n* = 4)	0.130	16.7% (*n* = 15)	9.1% (*n* = 1)	0.516
**Intraoperative data**						
Duration of surgery (min)	162.48 ± 51.35	182.29 ± 48.72	0.037*	168.39 ± 53.10	179.55 ± 63.74	0.521
Duration of anesthesia (min)	196.98 ± 55.24	215.88 ± 51.84	0.063	202.94 ± 56.44	217.00 ± 70.19	0.450
Blood loss (mL)	273.86 ± 165.21	324.52 ± 205.10	0.130	285.50 ± 184.74	281.82 ± 172.15	0.950
Blood transfusion	25.0% (*n* = 23)	42.9% (*n* = 18)	0.037*	31.1% (*n* = 28)	27.3% (*n* = 3)	0.794

Values are reported as mean ± standard deviation and numbers with percentages. The *P*-value is calculated by the independent-samples *T*-test, chi-square test, respectively. *p < 0.05 compared with group Non-PND_1_. **^+^***p* < 0.05 compared with group Non-PND_2_.

BMI, Body Mass Index; ASA, American Society of Anesthesiology.

**TABLE 2:** Neuropsychological test results for patients with/without PND.

	Time	Non-PND	PND	*T*-value	*P*-value
MMSE	Preoperative day 1	25.20 ± 1.51	25.17 ± 1.08	0.112	0.911
	Postoperative day 7	25.27 ± 1.68	22.64 ± 1.27	9.041	0.000*
	Preoperative day 1	25.16 ± 1.45	25.00 ± 1.34	0.339	0.735
	Postoperative day 90	25.21 ± 1.43	23.00 ± 1.55	4.788	0.000**^+^**
MoCA	Preoperative day 1	20.54 ± 3.23	20.12 ± 3.28	0.703	0.483
	Postoperative day 7	20.54 ± 3.39	18.50 ± 3.19	3.296	0.001*
	Preoperative day 1	20.13 ± 3.30	18.55 ± 3.59	1.493	0.139
	Postoperative day 90	20.48 ± 3.71	17.82 ± 3.97	2.227	0.028**^+^**

Pre-/Post-operative MMSE and MoCA scores for patients with/without PND are presented as mean ± standard deviation. The *P*-value is calculated by independent-samples *T*-test. *p < 0.05 compared with group Non-PND_1_. **^+^***p* < 0.05 compared with group Non-PND_2_.

### Preoperative heterogeneous nuclear ribonucleoprotein A2/B1 level increases in patients with postoperative neurocognitive dysfunction

**FIGURE 2:**
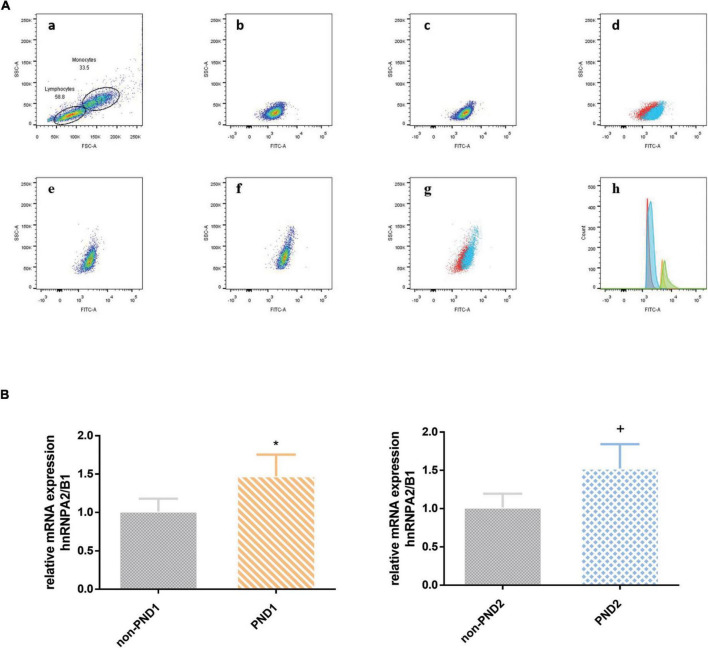
**(A)** The expression of hnRNPA2/B1 in PBMC was analyzed by FCM. The effect regions were created for FSC and SSC **(a)**; median fluorescence intensity of hnRNPA2/B1 in lymphocytes between PND and non-PND **(b,c)**, and the overlay of two groups (**d**; PND, blue; non-PND, red); median fluorescence intensity of hnRNPA2/B1 in monocytes between PND and non-PND **(e,f)**, and the overlay of two groups (**G**; PND, blue; non-PND, red); histogram overlay of hnRNP A2/B1 expression in patients with PND compared to patients without PND in different cell population (**h**; lymphocytes of non-PND, red; lymphocytes of PND, blue; monocytes of non-PND, orange; monocytes of PND, green). **(B)** mRNA level analysis of hnRNPA2/B1 in PBMCs. Data are expressed as the mean ± SD. Note that mRNA expression of hnRNPA2/B1 in patients with PND significantly increased compared with those without PND. *p < 0.05 compared with group Non-PND_1_. **^+^***p* < 0.05 compared with group Non-PND_2_.

**FIGURE 3:**
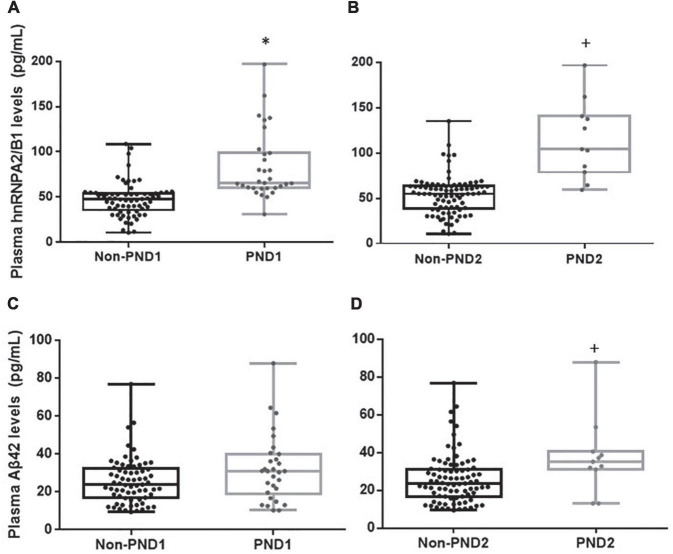
Plasma hnRNPA2/B1, Aβ_42_. Concentrations of hnRNPA2/B1 or Aβ_42_ in patients with or without PND at 7 days **(A,C)** and 3 months **(B,D)** after surgery. Boxes represent the median, the 25th and 75th percentiles, and bars indicate the range of data distribution. *p < 0.05 compared with group Non-PND_1_.^+^*p* < 0.05 compared with group Non-PND_2_.

Both the median fluorescence intensity and mRNA of preoperative hnRNPA2/B1 levels in the PND group were significantly higher than the other one. Besides, the median fluorescence intensity of hnRNPA2/B1 in monocytes was higher than that in lymphocytes (Figure 2). The preoperative hnRNPA2/B1 and Aβ_42_ levels in the PND group were significantly higher than that in the non-PND group, except for preoperative Aβ_42_ level at 7 days after surgery (Figure 3 and Table 3 *P* < 0.05). Moreover, we analyzed the sex differences of two biomarkers, hnRNPA2/B1 as well as Aβ_42_, and found no sex differences in both plasma biomarkers (Supplementary Table).

**TABLE 3:** Plasma hnRNPA2/B1 and Aβ_42_ levels for patients with/without PND [pg/mL, M (Q1, Q3)].

Time	Variable	Non-PND	PND	*Z*-value	*P*-value
Postoperative day 7	hnRNPA2/B1	54.95 (37.23, 63.35)	72.26 (58.33, 133.32)	−2.292	0.022*
	Aβ_42_	22.62 (12.94, 30.21)	30.78 (21.33, 45.77)	−1.230	0.219
Postoperative day 90	hnRNPA2/B1	56.38 (40.06, 64.99)	102.93 (59.46, 140.78)	−2.525	0.012**^+^**
	Aβ_42_	23.98 (16.80, 31.48)	35.23 (31.13, 40.61)	−2.453	0.014**^+^**

Data are presented as median with inter-quartile range. The *P*-value is calculated by the Mann-Whitney *U*-test. *p < 0.05 compared with group Non-PND_1_. **^+^***p* < 0.05 compared with group Non-PND_2_.

### Heterogeneous nuclear ribonucleoprotein A2/B1 is able to early predict the occurrence of postoperative neurocognitive dysfunction and has high diagnostic efficiency

**FIGURE 4:**
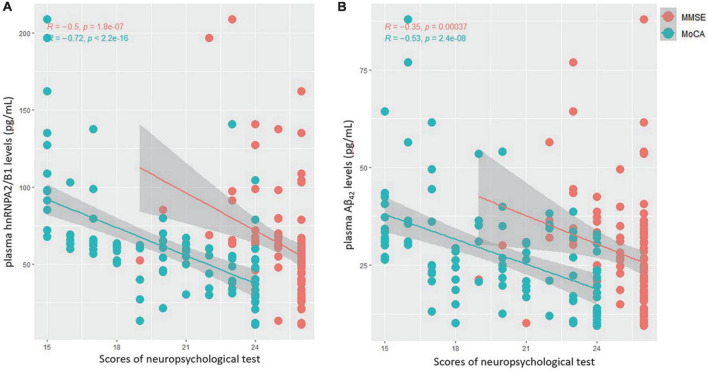
**(A)** Correlations between plasma hnRNPA2/B1 levels and MMSE(red) as well as MoCA(green) scores. **(B)** Correlations between plasma Aβ_4_2 levels and MMSE(red) as well as MoCA(green) scores.

As shown within Figure 4, univariate linear regression analysis showed that hnRNPA2/B1 had a stronger negative correlation with preoperative MoCA scores (*R* = −0.72, *p* < 0.001) than Aβ_42_ (*R* = −0.53, *p* < 0.001). The similar results could also be found in the correlation with preoperative MMSE scores (*R* = −0.50 in hnRNPA2/B1, *R* = −0.35 in Aβ_42_, *p* < 0.001).

**FIGURE 5:**
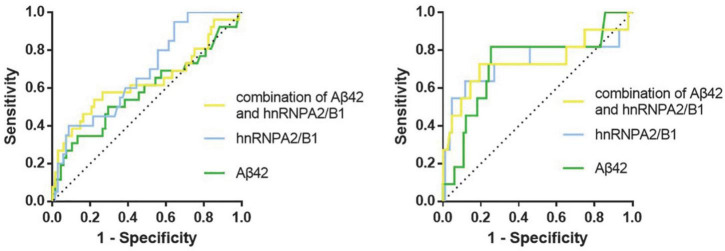
ROC curves for preoperative biomarkers. The figure showed the ROC curves for hnRNPA2/B1, Aβ_42_ and their combination at 7 days **(Left)** and 3 months **(Right)** after surgery, respectively.

**FIGURE 6:**
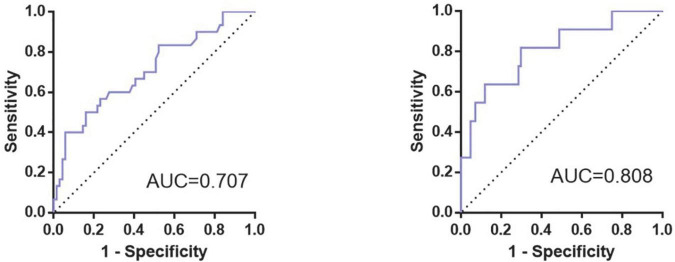
ROC curve for prediction model of occurrence of PND. The figure showed the ROC curves for the prediction model of PND at 7 days (Left) and 3 months (Right) after surgery, whose area under the curve (AUC) were 0.707 and 0.808, respectively.

According to the ROC curve of PND_1_, the cut-off value (59.26 pg/mL, sen: 0.687, spe: 0.649) of preoperative hnRNPA2/B1 level was determined by Youden Index, and the AUC was 0.686 (95% CI 0.568∼0.804). The cut-off value (30.48 pg/mL, sen: 0.567, spe:0.714) of preoperative Aβ_42_ level was determined by Youden Index, and the AUC was 0.616 (95% CI 0.489∼0.744). In PND_2_, the cut-off value (75.53 pg/mL, sen:0.882, spe:0.636) of preoperative hnRNPA2/B1 level was determined by Youden Index, and the AUC was 0.735 (95% CI 0.525∼0.945). The cut-off value (31.10 pg/mL, sen:0.818, spe:0.747) of preoperative Aβ_42_ level was determined by Youden Index, and the AUC was 0.728 (95% CI 0.554∼0.903). In addition, combination hnRNPA2/B1 and Aβ_42_ could further increase the specificity of predictive value but the sensitivity of model did not improve. In the longer follow-up duration, the efficacy of these two biomarkers and their combination all increased a lot (Figure 5 and Table 4). To determine whether preoperative hnRNPA2/B1 level was an independent risk factor for PND, a multivariable logistic regression analysis was performed. The results showed that the preoperative hnRNPA2/B1 level was an independent risk factor of PND (Table 5). Finally, the model’s predictive effect was examined by the ROC curve (Figure 6).

**TABLE 4:** ROC curve analysis results of plasma hnRNPA2/B1 and Aβ_42_ levels.

Time	Variable	Sensitivity	Specificity	Youden index	AUC	95% CI
Postoperative day 7	hnRNPA2/B1	0.687	0.649	0.336	0.686	0.568∼0.804
	Aβ_42_	0.567	0.714	0.281	0.616	0.489∼0.744
	hnRNPA2/B1 + Aβ_42_	0.567	0.783	0.349	0.664	0.538∼0.791
Postoperative day 90	hnRNPA2/B1	0.882	0.636	0.519	0.735	0.525∼0.945
	Aβ_42_	0.818	0.747	0.565	0.728	0.554∼0.903
	hnRNPA2/B1 + Aβ_42_	0.727	0.807	0.535	0.738	0.535∼0.940

**TABLE 5:** Multivariate logistic regression analysis.

Factors	PND_1_		PND_2_	
	Odds ratio (95% CI)	*P*-value	Odds ratio (95% CI)	*P*-value
Blood transfusion	2.066 (0.747∼5.717)	0.162	0.899 (0.169∼4.796)	0.901
Education level	0.932 (0.837∼1.038)	0.200	0.948 (0.805∼1.117)	0.525
hnRNPA2/B1	1.018 (1.003∼1.034)	0.018*	1.023 (1.007∼1.039)	0.005*
Aβ_42_	1.021 (0.987∼1.056)	0.237	1.033 (0.992∼1.077)	0.120
DM	1.139 (0.383∼3. 384)	0.815	2.039 (0.430∼9.680)	0.370
Duration of surgery	1.003 (0.994∼1.012)	0.475	1.003 (0.992∼1.077)	0.734
Age	1.031 (0.923∼1.152)	0.587	0.972 (0.812∼1.163)	0.755

*P means *P*-value < 0.05.

OR, Odds ratio; CI, Confidence interval.

## Conclusion

Preoperative higher level of hnRNPA2/B1 is relevant to a higher risk of PND in patients after lumbar decompression and fusion. Therefore, it seems that hnRNPA2/B1 could be a clinically valuable biomarker to predict PND and patients with a higher preoperative hnRNPA2/B1 should be placed more emphasis. Considering that the number of the elderly who need surgical therapy is growing, more extensive clinical and basic studies are urgently needed to ensure our findings as well as further clarify the potential mechanisms.

## Discussion

We identified PND in 31.3% of patients at 7 days after lumbar decompression and fusion and 10.9% at 3 months after surgery. Patients with higher preoperative hnRNPA2/B1 level tend to present cognitive decline for a more extended time after operation (median, 72.26 pg/mL in PND_1_, 102.93 pg/mL in PND_2_). High hnRNPA2/B1 was a significant predictor of PND and the cut-off value for hnRNPA2/B1 was 75.53 pg/mL (AUC, 0.735; 95% CI 0.525∼0.945), whose predictive value could improve further with the consideration of individual demographic data at baseline. Therefore, hnRNPA2/B1 is expected to become a new and powerful predictive marker to identify elderly patients with PND.

Current theories on the etiology of PND now include surgery-related factors such as surgery time, patient position and postoperative infection; anesthesia-related factors such as anesthesia method and intraoperative hypotension; and patient-related factors such as age and physical state. By analyzing several basic characteristics of enrolled patients, we found that there was significant difference in history of intraoperative blood transfusion, educational attainment, preoperative comorbidity, and the duration of surgery and anesthesia.

It’s generally believed that educational attainment can predict postoperative neurocognitive disorders to some degree, an effect that can be attributed to cognitive reserve as well as brain connectivity (Monk et al., 2008; Arenaza-Urquijo et al., 2013; Feinkohl et al., 2017b; Perry et al., 2017), which is in line with our findings. And we observed no clinically meaningful difference in age though there is no doubt that aging itself is the major independent risk factor for age-associated disorders. We think that’s because the age distribution of patients enrolled in our study mainly concentrated in the narrow age group of 65∼70 years old, some survey about the shifting architecture of cognition across the adult life span shows that compared to other age groups, there is no significant decline in long-term memory and working memory between 60 and 70 years old (Park and Reuter-Lorenz, 2009).

We also investigated the association of some preoperative comorbidities with the risk of PND, such as hypertension, diabetes, coronary artery heart disease. We found significant association for diabetes but not for hypertension or coronary artery heart disease with PND risk, which is consistent with the results of some studies (Feinkohl et al., 2017a,^2018^; Lachmann et al., 2018). Several lines of evidence suggest that its possible mechanism may be the changes in brain structure and function, such as impaired glucose metabolism, cerebral atrophy, cerebrovascular damage (Moran et al., 2019; van Sloten et al., 2020; Krell-Roesch et al., 2021). As for other preoperative complications, our findings differ from some research that a range of cardiovascular conditions, including hypertension and coronary artery heart disease, would attribute to cognitive dysfunction induced by cerebral small vessel lesions (Liu et al., 2018; Iadecola and Gottesman, 2019). We consider that is because majority of participants could take medicine punctually to monitor their blood pressure regularly, and antihypertensive agents are significantly associated with a lower risk of dementia or cognitive impairment (Hughes et al., 2020; de Menezes et al., 2021). Of course, there is another possibility that we should have assessed the severity of hypertension and duration of diagnosis which may be important to cognitive risk prediction.

In terms of surgical information, we analyzed the relationship of operative duration and history of intraoperative blood transfusion with PND. Lumbar decompression and fusion is a major operation with extensive trauma, long duration and large amount of intraoperative blood loss, resulting in various postoperative complications (Heemskerk et al., 2021). Blood transfusion is a lifesaving treatment, which can provide volume expansion and increase oxygen-carrying capacity. Nevertheless, transfusion of allogeneic blood products can also trigger enhanced acute inflammatory responses (Cata et al., 2013). We showed that history of intraoperative blood transfusion may contribute to short-term cognitive decline after surgery, while not to long-term cognitive performance. Several lines of findings show that some inflammatory factors would remarkably elevate due to blood transfusion, such as tumor necrosis factor (TNF)-α, interleukin (IL)-β, IL-6, and IL-8, and then lead to neurocognitive impairment (Urner et al., 2012; Ferraris et al., 2013). In addition, transfusion of old red blood cells (>14 days) frequently occurs in clinical practice, inducing neuroinflammation and the development of PND *via* cell-free hemoglobin (Zhu et al., 2014; Tan et al., 2015). Surgery-induced cognitive decline may be attributed to the microglial activation, and the latter would result the release of inflammatory factors and neuroendocrine hormone (Terrando et al., 2011; Subramaniyan and Terrando, 2019; Xiao et al., 2020). Therefore, longer duration of operation is associated with a stronger inflammatory response. Moreover, we found this perioperative stress doesn’t seem to cause long-term cognitive deficit.

Apart from paying attention to the baseline information and clinical data, we also explored the biomarkers of PND, aiming to provide guidance for clinical perioperative brain protection, and even promote the screening of patients before admission to hospital and facilitate the process of brain health.

PND has been demonstrated to be associated with multiple factors, among which the theories of neuroinflammation and oxidative stress may be the mainstream. In recent years, there are mounting studies about the association between hnRNPA2/B1 and the exacerbation of cognitive performance (Mizukami et al., 2005; Shorter and Taylor, 2013; Lorente Pons et al., 2020). HnRNPA2/B1 could affect cognitive function through alternative splicing and hence lead to the over-expression of β-secretase 1, and which could increase the accumulation of amyloid precursor protein and soluble Aβ_1–42_ in cytoplasm and promote the phosphorylation of tau protein (Kolisnyk et al., 2017; Zhang et al., 2021). These pathological changes not only damage the neurons directly, but also indirectly trigger neuroinflammation by interacting synergistically with glial cells, ultimately making neurodegenerative lesion stabile and irreversible. What’s more, the prion-like domains (PrLD) of hnRNPA2/B1 are the critical components that drive liquid–liquid phase separation (LLPS) and subsequently contribute stress granule (SG) to the hydrogel phase transition (Lee et al., 2016), while the latter is the key mechanism of conversion from neurodegeneration to dementia in the elderly (Lu et al., 2020; Rossi et al., 2020). Consistently, we found that the elderly with PND usually had a higher preoperative hnRNPA2/B1 level and this biomarker could provide preferable specificity and sensitivity to predict PND. In summary, our finding that hnRNPA2/B1 combined with Aβ_42_ could further increase the predictive value is encouraging as it could accurately identify people at high risk of PND.

As for the logistic multivariable regression and the prediction model, we only took preoperative hnRNPA2/B1 and Aβ_42_ level, intraoperative blood transfusion event, educational level, diabetes, duration of surgery and age into consideration due to the size of the sample, the former six factors are significantly different in our single variable analysis. While age has been reported as comorbidities of PND in previous literature (Monk et al., 2008; Wu et al., 2016). With the result, we built a prediction model for PND, and its AUC of the ROC curve was 0.707 in short-term follow-up and 0.808 in a longer time. As a result, this model is meaningful to predict PND after major orthopedic surgery at the early stage, guiding proper care for the patients at high-risk of PND much earlier. Though there is a prediction model of postoperative cognitive outcomes for elderly orthopedic patients, this model was based on postoperative delirium only at 1 day after surgery (Wang et al., 2021). Therefore, our research is novel and profound with the consideration of long-term cognitive changes after surgery, while a larger scale of multicenter clinical researches need to be conducted in the future.

For the first time to explore the influence of hnRNPA2/B1 on the occurrence of PND after spinal surgery, our finding could give clinicians assistance to monitor and intervene the cognitive function in the elderly patients at the early stage. Sleep, pain, and cognition are three key intervenable targets in any multicomponent intervention designed to optimize perioperative brain (O’Gara et al., 2021). In consequence, we could reduce the occurrence and severity of PND by week-long cognitive training before surgery, relieving preoperative pain and tension, improving the sleeping conditions and so on (Ball et al., 2002; Sieber et al., 2011; Kamdar et al., 2013).

There are several limitations in our present study. Firstly, patients are usually discharged from hospital within 5 days, making it hard to collect blood samples in a longer follow-up period and investigate the dynamic changes of hnRNPA2/B1 in the development of disease as well as the severity of cognitive decline. Moreover, we only observed cognitive outcomes at 7 days and 3 months after surgery and did not perform longer follow-up. Therefore, longer-term impact of preoperative hnRNPA2/B1 level to AD conversion remains unknown. In the future, we will continue to follow up this population to clarify the relationship between hnRNPA2B1 and the outcome of PND and establish a complete perioperative warning system of MCI-PND-AD. A large-scale multicenter study will be carried out to further validate our findings as well. However, it should be noted that, we have indeed found some patients present a tendency to progress to dementia at the 3-month follow-up after surgery, whose neuropsychological performance is poorer than before and meet the criteria of dementia.

Briefly, our data expand the clinical value of hnRNPA2/B1 and suggest that patients occurring PND after lumbar decompression and fusion have higher hnRNPA2/B1 level as compared to controls. When taking baseline characteristics and biomarker signature of patients into account, we believe that increasing number of individuals at high risk of PND will be identified with high accuracy for they may benefit the most from timely interventions aimed at preventing pathological cognitive decline.

## Data availability statement

The datasets presented in this study can be found in online repositories. The names of the repository/repositories and accession number(s) can be found in the article/Supplementary material.

## Ethics statement

The studies involving human participants were reviewed and approved by the Medical Ethics Committee of Tianjin Third Central Hospital Nankai University Affinity the Third Central Hospital. The patients/participants provided their written informed consent to participate in this study.

## Author contributions

HW: study design and manuscript revision. TX: data analysis and manuscript writing. CY: FCM. XW: follow-up of patients and post-operative data acquisition. LB: subjects’ recruitment. JM: qRT-PCR. MZ: ELISA. WH: peri- and intra-operative data acquisition. All authors contributed to the article and approved the submitted version.
