# Supplementary material for: Heterogeneous nuclear ribonucleoprotein A2/B1 as a novel biomarker in elderly patients for the prediction of postoperative neurocognitive dysfunction: A prospective nested case-control study
Source: Front Aging Neurosci. 2022 Oct 21;14:1034041. doi: 10.3389/fnagi.2022.1034041 (PMC9634074; doi:10.3389/fnagi.2022.1034041)
Supplement: Supplementary file 1 [file Table_1.DOCX]

**Suppl Table 1. Plasma hnRNPA2/B1 and Aβ42 levels shown by sex [pg/mL, M(Q1, Q3)]**

| biomarker | male | female | Z value | P value |
| --- | --- | --- | --- | --- |
| hnRNPA2/B1 | 54.95 (37.79, 65.35) | 61.92 (46.89, 71.08) | -1.856 | 0.063 |
| Aβ_42_ | 23.30 (15.80, 30.32) | 29.33 (17.88, 35.91) | -1.752 | 0.080 |

Data are presented as median with inter-quartile range. The P-value is calculated by the Mann-Whitney U test.
